# Emergency Endovascular Repair for the Non-anastomotic Rupture of a 14-Year-Old Reverse Lock-Knitted Dacron Graft

**DOI:** 10.7759/cureus.82925

**Published:** 2025-04-24

**Authors:** Kensuke Kobayashi, Yusuke Mizuno, Rina Suzuki

**Affiliations:** 1 Cardiac Surgery, Daiyukai General Hospital, Ichinomiya, JPN

**Keywords:** abdominal emergency medicine, dacron graft, endovascular repair, non-anastomotic rupture, vascular endoprosthesis

## Abstract

An 80-year-old man who underwent abdominal aortic replacement 14 years earlier collapsed suddenly and was rushed into our emergency room. In a state of confusion on arrival, he ended up experiencing cardiopulmonary arrest. Cardiopulmonary resuscitation was successful, and a contrast-enhanced computed tomography revealed a massive retroperitoneal hemorrhage around the bifurcated prosthetic graft. Aortography revealed a non-anastomotic rupture of the graft’s body. An emergency endovascular repair was successfully performed; however, the patient died due to disseminated intravascular coagulation and multiple organ failure on postoperative day one.

## Introduction

Dacron prosthetic grafts, which are known to be durable, have been widely used for aortic surgery. However, cases of late non-anastomotic rupture or aneurysm formation of an intrinsic Dacron prosthesis have been reported [1-7]. In recent years, under the technological advancement of the material and sealing methodology of the knitted Dacron prosthesis, there has been a paucity of reports of intrinsic graft failure. Herein, we report a rare case of emergency endovascular repair of a late non-anastomotic rupture of a modern bifurcated knitted Dacron prosthesis, i.e., Intergard Knitted Vascular Graft (IKVG) (GETINGE AB, Göteborg, Sweden).

## Case presentation

**Figure 1:**
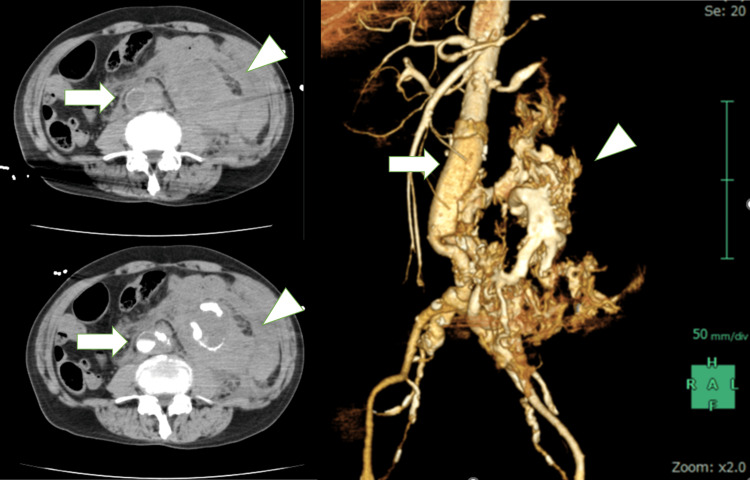
Emergency contrast-enhanced computed tomography A contrast-enhanced computed tomography scan revealed massive retroperitoneal hemorrhage (arrowheads) around the bifurcated graft (arrows). There was no residual or recurrent aneurysm. It was difficult to identify the bleeding site.

An 80-year-old man with chronic kidney disease on hemodialysis collapsed suddenly while going out and was rushed into our emergency room. He had previously undergone abdominal aortic replacement using a 16 × 8 mm bifurcated IKVG (IGK1608) for an infrarenal abdominal aortic aneurysm at another hospital 14 years earlier. Moreover, two years previously, he received intensity-modulated radiotherapy (IMRT) at a total dose of 76 Gy for prostate cancer. On arrival, he was in a state of confusion with his systolic blood pressure at 40-60 mmHg, ultimately ending up in cardiopulmonary arrest. After successful cardiopulmonary resuscitation, contrast-enhanced computed tomography angiography revealed massive retroperitoneal hemorrhage around the bifurcated prosthetic graft; however, the bleeding site remained unclear (Figure 1).

**Figure 2:**
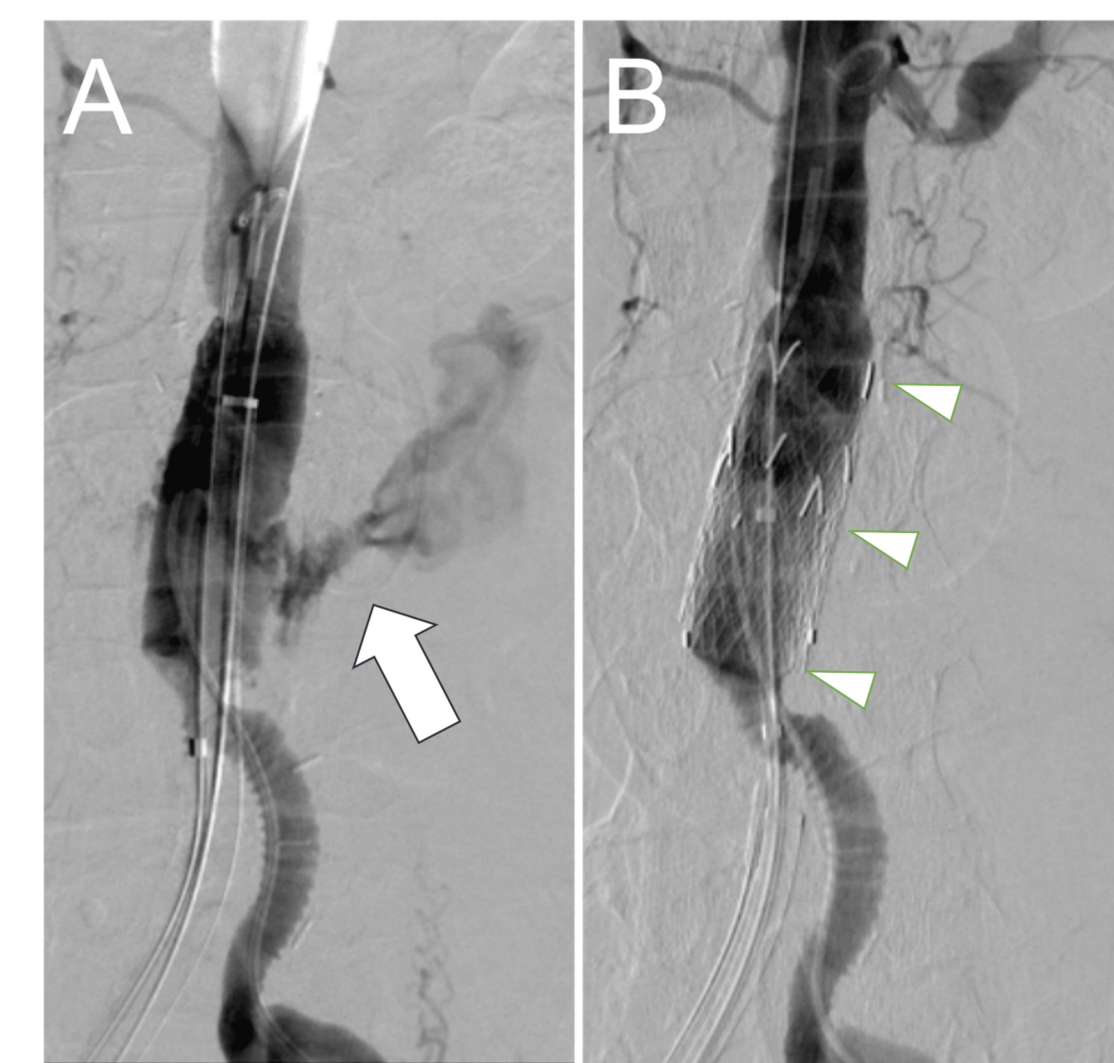
Emergency endovascular repair of the ruptured Dacron graft Emergency catheter aortography revealed a non-anastomotic rupture of the prosthetic graft’s midbody (arrow) (A). The measured diameter of the graft’s body was 22 mm, and the length from the proximal anastomosis to the graft bifurcation was 66 mm. Complete hemostasis was achieved using three overlapped pieces of 23-mm-diameter and 33-mm-long straight stent grafts (arrowheads) (B).

After introducing an intra-aortic balloon occlusion for temporary hemostasis, catheter aortography revealed a non-anastomotic rupture of the midbody of the prosthetic graft (Figure 2A). Angiographically, the diameter of the graft’s body was 22 mm, and the distance from the proximal anastomosis to the graft bifurcation was 66 mm. An emergency endovascular repair performed using three pieces of 23-mm-diameter and 33-mm-long GORE EXCLUDER Aortic Extender Endoprosthesis (PLA230300) (W. L. Gore & Associates, Inc., Flagstaff, AZ, USA) was successfully completed (Figure 2B). That notwithstanding, the patient died due to disseminated intravascular coagulation and multiple organ failure on postoperative day one. A pathological autopsy was not performed as the family did not consent.

## Discussion

A late non-anastomotic rupture of the Dacron prosthetic graft is a sudden and devastating adverse event. It is a rare but well-known life-threatening phenomenon commonly reported as a complication of the knitted Dacron prosthesis rather than the woven one [1,2]. Since the early 1980s, it has been pointed out that late fiber deterioration of the Dacron prosthesis can occur due to various factors, such as manufacturing problems, storage conditions, sterilization methods, intraoperative handling, and bio-degradative effects of tissue fluids or enzymes [3]. In many cases, intrinsic rupture or aneurysm formation of the Dacron prosthesis occurs more than a decade after surgery, and in some cases, it occurs more than three decades thereafter [2-5]. Akiyama et al. reported a rare case of pseudoaneurysm formation due to bleeding through the fiber interstices without rupture of a knitted Dacron graft 12 years after surgery [1].

The IKVG, one of the durable and easy-to-handle knitted Dacron grafts, applies bovine collagen coating and reverse lock-knit technology. The reverse lock-knit technology aims to improve the anti-dilatation properties of the knitted Dacron prosthesis, and compared to the conventional wrap-knitted Dacron prosthesis, the dilatation rate was significantly suppressed over a five-year period [6]. Although the risk of late graft deterioration of IKVG may have been mitigated, Miszczuk et al. reported a case of rapid graft dilatation of the 22-mm-diameter IKVG, which consequently reached a diameter of 47 mm during their five-year follow-up period [7]. In the present report, we described a rare case of the late non-anastomotic rupture of a bifurcated IKVG 14 years postoperatively. Because no pathological autopsy was performed, the cause of the graft rupture is unclear. The patient had previously undergone IMRT at a total dose of 76 Gy for prostate cancer. However, IMRT is a precise radiation therapy modality aimed at prostate cancer while protecting the healthy surrounding tissue. Moreover, Seydel et al. reported that radiation in the therapeutic dose range (10-80 Gy) did not affect the tensile strength and elongation of the Dacron prosthesis [8]. It is unlikely that IMRT induced the deterioration or rupture of the Dacron prosthesis.

Endovascular repair for the ruptured main body of a bifurcated prosthetic graft is profitable in terms of rapid emergency response. However, effective hemostasis performed using straight stent grafts requires a sufficient distance from the renal arteries to the graft bifurcation. In general, abdominal aortic replacement with a bifurcated prosthesis is likely to shorten the graft’s main body. This means that deploying commercially available straight or bifurcated stent grafts while avoiding renal artery coverage might be challenging; therefore, aorto-uni-iliac stent grafting with concomitant femoral-femoral crossover bypass must be performed when considering endovascular repair with current devices [9]. In this report, the length of the main body was sufficient to deploy short and straight stent grafts. Three non-barbed stent graft pieces were overlapped to achieve complete hemostasis.

## Conclusions

Herein, we report a rare case of emergency endovascular repair of a late non-anastomotic rupture of the IKVG. In recent years, due in part to advances in material technology, there have been few reports of failure or deterioration of Dacron prosthetic vascular grafts. However, even with modern vascular prostheses, late graft deterioration and rupture are life-threatening problems.

We achieved complete hemostasis using endovascular procedures with three overlapped pieces of 23-mm-diameter and 33-mm-long straight stent grafts. Although etiological findings were not obtained, describing the procedures performed for rapid hemostasis and its pitfalls may contribute to similar cases.
